# Protective Effect of Klotho against Ischemic Brain Injury Is Associated with Inhibition of RIG-I/NF-κB Signaling

**DOI:** 10.3389/fphar.2017.00950

**Published:** 2018-01-18

**Authors:** Hong-Jing Zhou, Hui Li, Meng-Qi Shi, Xiao-Na Mao, Dong-Ling Liu, Yi-Ran Chang, Yu-Miao Gan, Xi Kuang, Jun-Rong Du

**Affiliations:** Department of Pharmacology, Key Laboratory of Drug Targeting and Drug Delivery System, West China School of Pharmacy, Sichuan University, Chengdu, China

**Keywords:** Klotho, ischemic stroke, neuroinflammation, RIG-I/NF-κB signaling, aging

## Abstract

Aging is the greatest independent risk factor for the occurrence of stroke and poor outcomes, at least partially through progressive increases in oxidative stress and inflammation with advanced age. Klotho is an antiaging gene, the expression of which declines with age. Klotho may protect against neuronal oxidative damage that is induced by glutamate. The present study investigated the effects of Klotho overexpression and knockdown by an intracerebroventricular injection of a lentiviral vector that encoded murine Klotho (LV-KL) or rat Klotho short-hairpin RNA (LV-KL shRNA) on cerebral ischemia injury and the underlying anti-neuroinflammatory mechanism. The overexpression of Klotho induced by LV-KL significantly improved neurobehavioral deficits and increased the number of live neurons in the hippocampal CA1 and caudate putamen subregions 72 h after cerebral hypoperfusion that was induced by transient bilateral common carotid artery occlusion (2VO) in mice. The overexpression of Klotho significantly decreased the immunoreactivity of glial fibrillary acidic protein and ionized calcium binding adaptor molecule-1, the expression of retinoic-acid-inducible gene-I, the nuclear translocation of nuclear factor-κB, and the production of proinflammatory cytokines (tumor necrosis factor α and interleukin-6) in 2VO mice. The knockdown of Klotho mediated by LV-KL shRNA in the brain exacerbated neurological dysfunction and cerebral infarct after 22 h of reperfusion following 2 h middle cerebral artery occlusion in rats. These findings suggest that Klotho itself or enhancers of Klotho may compensate for its aging-related decline, thus providing a promising therapeutic approach for acute ischemic stroke during advanced age.

## Introduction

Ischemic stroke is a leading cause of severe disability, with high morbidity that accounts for 11.13% of total deaths worldwide (Mozaffarian et al., 2015). Aging has been shown to be the greatest independent risk factor for the occurrence of stroke and poor outcomes (Rosen et al., 2005; Aurel et al., 2007). The number of stroke incidents is expected to more than double from 2010 to 2050, with the majority of this increase among older adults who are more than 75 years old and a 100-fold greater post-stroke mortality risk among the elderly compared with adults who are 25–44 years old (Troen, 2003). Although age-related pathophysiological changes in the brain are very complicated, chronic low-grade neuroinflammation and an increase in oxidative stress have been shown to be highly associated with the impairment of ischemic resistance in the aging brain (Calabrese et al., 2007; DiNapoli et al., 2008). However, the underlying molecular mechanisms remain unclear.

Klotho is an antiaging gene with pleiotropic effects. It is reported to be profoundly involved in the aging process. Mice that were Klotho-deficient developed human-like premature aging phenotypes, including multiple organ failure, cognitive impairment, and a shortened life span, which could be rescued by Klotho overexpression (Kuro-o et al., 1997; Kurosu et al., 2005). Klotho may encode a type I transmembrane protein that is highly expressed in the cerebral choroid plexus and renal tubular epithelia with a large extracellular domain and a short intracellular portion (Kuro-o et al., 1997). During processing by secretases, Klotho may be cleaved into extracellular and intracellular forms (Imura et al., 2004; Chen et al., 2007; Bolch et al., 2009). The shed ectodomain of Klotho is released into cerebrospinal fluid (CSF) and blood, exerting potent antioxidative activity (Zeldich et al., 2014). Intracellular Klotho is the cytoplasmic C-terminus after cleavage, and may act as an endogenous inhibitor of retinoic-acid-inducible gene-I (RIG-I)-mediated inflammation (Liu et al., 2011). Several studies have reported the anti-inflammatory effects of Klotho in cultured cells and an association between a reduction of Klotho and the inflammatory response in chronic obstructive pulmonary disease and renal injury (Maekawa et al., 2009; Li et al., 2015; Xia et al., 2016; Zeng et al., 2016). Moreover, Klotho has been identified as a genetic risk factor for ischemic stroke that is caused by cardioembolism in Korean females (Kim et al., 2006).

Preclinical and clinical data indicate the age-related decline of Klotho expression in the brain. Cerebral Klotho is lower in elderly than in young rhesus monkeys, and serum Klotho levels are inversely correlated with the age of subjects (Duce et al., 2008; Yamazaki et al., 2010; King et al., 2012b; Pedersen et al., 2013). Our previous study showed that the levels of Klotho mRNA and protein significantly decreased in the cerebral choroid plexus in aging mice (Kuang et al., 2014a). We hypothesized that the loss of endogenous Klotho with advanced age increases the susceptibility of brain cells to ischemia through the induction of oxidative stress and neuroinflammation and worsens neurological outcomes in senile stroke patients. Zeldich et al. (2014) reported a direct protective effect of Klotho protein against neuronal oxidative damage that was induced by glutamate. The present study explored the potential role of Klotho in ischemic brain injury in rodents. Our results showed that the upregulation of cerebral Klotho expression via gene delivery significantly protected against ischemic brain injury in mice with forebrain ischemia. This protective effect was attributable to the induction of RIG-I/nuclear factor-κB (NF-κB) signaling inhibition. In addition, the knockdown of Klotho in the brain exacerbated neurological dysfunction and cerebral infarct in rats with transient focal cerebral ischemia.

## Materials and Methods

### Animals

Specific-pathogen-free male C57BL/6 mice (6 weeks old, weighing 20–22 g) and Wistar rats (3 months old, weighing 320–350 g) were supplied by Chongqing Tengxin Experimental Animals Co. Ltd. and Chengdu Dossy Experimental Animals Co. Ltd. (Chengdu, Sichuan, China), respectively. All of the animals were housed in stainless-steel cages at 21°C ± 2°C and had free access to food and water. The animals were housed under a 12 h/12 h light/dark cycle (lights on 7:00 AM-7:00 PM). The animal studies were conducted in accordance with the Regulations of Experimental Animal Administration issued by the State Committee of Science and Technology of the People’s Republic of China. All of the procedures were approved by the Animal Research Committee of West China School of Pharmacy.

### Regulation of Klotho Expression by Lentivirus in the Brain

#### Lentivirus Production

Lentiviral vectors that encoded the transmembrane form of mouse Klotho (LV-KL) and harbored a short-hairpin RNA sequence that targeted rat Klotho (LV-KL shRNA) and corresponding control lentiviral vectors that encoded green florescence protein (LV-GFP) were obtained from OriGene (Rockville, MD, United States). They were used to transform competent DH5α *Escherichia coli* bacterial strains for amplification, followed by extraction using the E.Z.N.A^TM^ plasmid mini kit (Omega, Norcross, GA, United States) and the production of lentiviral suspensions using the GM easy^TM^ lentiviral packaging kit (Genomeditech, Shanghai, China) according to the manufacturer’s instruction. When HEK293 cells (ATCC, Manassas, VA, United States) reached 90–100% confluence, GM easy^TM^ lentiviral mix (Genomeditech, Shanghai, China), lipofectamine 3000 (Invitrogen, Carlsbad, CA, United States), and LV-KL or LV-GFP were mixed in serum-free, antibiotic-free Dulbecco’s Modified Eagle Medium and then added to the culture dishes together with fresh culture medium. Culture medium that contained lentivirus was collected 72 h after transfection and then filtered at 0.45 μm. Lentiviral enrichment reagent (Genomeditech, Shanghai, China) was added to the virus suspension, kept at 4°C overnight, and centrifuged at 4000 × *g* for 25 min. After resuspension with sterile phosphate-buffered saline (PBS), serially diluted lentivirus was used to transduce HEK293 cells. Seventy-two hours later, the labeled HEK293 cells were counted to calculate the viral titer.

#### Overexpression of Klotho Expression in Murine Brain

Transfection of the Klotho gene was induced by a bilateral intracerebroventricular injection of LV-KL or LV-GFP in the lateral ventricles in mice. After anesthetizing the mice with chloral hydrate (400 mg/kg, i.p.), the head was positioned in a stereotaxic frame (SM-15, Narishige Scientific Instrument Laboratory, Tokyo, Japan). A 30-gauge needle (Shanghai Golden Globe Medical Devices Co., Ltd., Shanghai, China) was inserted into the lateral ventricle through a burr hole in the skull (stereotaxic coordinates: 1.0 mm near the midline, 0.2 mm posterior to bregma, and 2.5 mm below the skull). Gene transfection was induced by an infusion of 3 μl/site LV-KL or LV-GFP (2.1 × 10^7^ TU/ml) at a constant flow rate of 0.50 μl/min using a microinfusion pump (LO170-1A, Baoding Longer Precision Pump Co., Ltd., Baoding, China). Throughout the period of surgery and anesthesia, the animals were allowed to breathe spontaneously, and rectal temperature was maintained at 37°C with a feedback-regulated heating pad. Four weeks later, bilateral common carotid artery occlusion (2VO) was performed in these animals as described previously (Kuang et al., 2006).

#### Knockdown of Klotho Expression in Rat Brain

Rats were anesthetized and intracerebroventricularly administered lentiviral vectors as described previously (Kuang et al., 2009). In brief, 10 μl of LV-GFP or LV-KL shRNA (2.1 × 10^9^ TU/ml) was bilaterally injected in the lateral ventricles at a flow rate of 1 μl/min over 10 min. Seven days after the injection of the lentivirus suspension, three rats per group underwent brain sample collection to determine the levels of Klotho mRNA in the choroid plexus and Klotho protein in cerebrospinal fluid. After the rats were deeply anesthetized, CSF was collected from the cisterna magna, and the choroid plexus was isolated under a microscope and immersed in Trizol reagent (Invitrogen Life Technologies, Carlsbad, CA, United States), both of which were stored at -80°C until use. Additional rats were subjected to transient middle cerebral artery occlusion (MCAO) as described previously (Kuang et al., 2014b).

### Global Cerebral Ischemia-Induced Injury in Mice

#### Induction of Global Cerebral Ischemia

Four weeks after the intracerebroventricular administration of LV-GFP or LV-KL, the mice were randomly divided into sham, LV-GFP, and LV-KL groups (*n* = 9/group) and subjected to 2VO (Kuang et al., 2006). Anesthesia was induced with 400 mg/kg chloral hydrate (i.p.). Both common carotid arteries were exposed through a midline incision in the neck and temporarily clipped for 20 min with micro-serrefines. Following occlusion, the clips were removed to restore blood flow for recirculation. Sham-operated mice underwent the same surgical procedure, but the arteries were not occluded. Throughout surgery, the animals were kept on a thermostatically controlled warming plate to maintain body temperature at 37°C.

#### Examination of Neurobehavioral Deficits

Neurobehavioral testing was performed 24, 48, and 72 h after reperfusion following 2VO as described previously (Marutani et al., 2012). Neurobehavioral deficits in mice were evaluated by a 14-point scoring system: (1) grasping movement reflex (i.e., induction of catching reflex by running a small rod over the plantar surface of the paw): 0–4 points; (2) stop at the edge of a table: 0 or 1 point; (3) turning the head (i.e., turning the head when touching the ear from behind with a small rod): 0–2 points; (4) falling reflex (i.e., lifting the mouse by the tail and lowering it with the front legs toward the ground): 0 or 1 point; (5) spontaneous motility (i.e., moving behavior on a flat surface): 0–2 points; (6) circling behavior (i.e., locomotor behavior on a flat surface): 0 or 2 points; (7) pelt appearance (i.e., appearance of the animal’s coat): 0 or 1 point; (8) flight reaction (i.e., spontaneous behavior on a flat surface): 0 or 1 point. Higher scores indicated worse neurological function.

#### Collection of Brain Tissue Samples

After the neurobehavioral tests, the mice were anesthetized and transcardially perfused with saline. For the histopathological examinations, five mice from each group were perfused with 4% paraformaldehyde. The brains were removed, immersed in fixative for 48 h, embedded in paraffin, and microsectioned at a thickness of 5 μm. For the biological analysis, the brains from an additional four mice per group were rapidly removed, and the choroid plexus and left hemisphere were isolated for quantitative polymerase chain reaction (qPCR), and the right hemisphere was collected for Western blot analysis.

#### Nissl Staining

Nissl staining was used to detect 2VO-induced neuronal injury as reported previously (Shen et al., 2011), with minor modification. Serial coronal paraffin sections were cut (from bregma) at 0.5 ± 0.2 mm for the caudate putamen (CPu) and -1.94 ± 0.2 mm for the hippocampal region. The sections were then processed for 0.5% Cresyl violet staining (Schmid GmbH Co., Schwaigern, Germany). The severity of neuronal damage was blindly evaluated by counting surviving neurons in the hippocampal CA1 and CPu areas under a microscope (Nikon, Tokyo, Japan). For the semiquantitative analysis of the histochemical results, three sections from each brain, with each section containing the hippocampal CA1 area and three microscopic fields from the ischemic CPu, were digitized under a 40× objective. The data are presented as the number of neurons in the CA1 subregion and the number of neurons per field in the CPu (Kuang et al., 2008).

### Focal Brain Ischemia-Induced Injury in Rats

#### Induction of Focal Cerebral Ischemia

Seven days after the intracerebroventricular injection of LV-GFP or LV-KL shRNA, the rats were subjected to right MCAO using the intraluminal filament technique while monitoring cerebral blood flow (CBF) in the right middle cerebral artery (Mao et al., 2017). Two hours after MCAO, the thread was carefully withdrawn to establish reperfusion. Rats with both sufficient ischemia (∼20% of CBF baseline) and reperfusion (>80% of CBF baseline) were included in the study (*n* = 3/group).

#### Examination of Neurobehavioral Deficits

Neurobehavioral testing was performed 22 h after reperfusion following 2-h MCAO by two examiners who were blind to the experimental groups (Mao et al., 2017). Neurological impairment after ischemic insult was evaluated using a neurobehavioral test that was scored on a 5-point scale: 0 (no significant deficits), 1 (failure to extend left forepaw fully), 2 (circling to the left), 3 (falling to the left), 4 (inability to walk spontaneously combined with depressed levels of consciousness).

#### Determination of Infarct Volume

Twenty-four hours after the onset of MCAO, three rats from each group were euthanized after the neurobehavioral test by rapid decapitation. The brains were rapidly removed and stained with 2,3,5-triphenyltetrazolium chloride (TTC; Sigma–Aldrich, St. Louis, MO, United States) as described previously (Mao et al., 2017). The infarct volume measurement was performed by an investigator who was blind to the treatment groups. The results are expressed as a percentage of the total brain volume.

### Immunostaining Assays

Immunohistochemistry and fluorescence staining were performed to examine Klotho protein expression in the choroid plexus and glial activation in the hippocampal CA1 subregion in mice as previously described (Kuang et al., 2014a; Mao et al., 2017). The selected coronal sections were incubated with a respective antibody against Klotho (1:200; Transgene, Raleigh, NC, United States), glial fibrillary acidic protein (GFAP; 1:100; Wanleibio, Shenyang, Liaoning, China), or Iba-1 (1:500; Wako, Wakayama, Japan) at 37°C for 2 h and then at 4°C overnight. The sections were then incubated with a secondary antibody conjugated with or without *fluorescein* isothiocyanate (Boster Biological Technology, Wuhan, Hube, China), followed by nucleus counterstaining with hematoxylin or 4,6-diamidino-2-phenylindole (DAPI; Boster). For semiquantitative analysis of the immunostaining results, the choroid plexus or hippocampal CA1 region was digitized using a 40× objective. Immunoreactivity was determined based on the integrated optical density (IOD) of Klotho-positive immunostaining in the choroid plexus or the percent area of GFAP- and Iba-1-positive immunostaining in the CA1 subfield using Image Pro Plus 6.0.

### Western Blot Analysis

To examine target protein levels by Western blot, the nuclear protein of mouse brain tissues was isolated using RIPA buffer (Beyotime, Jiangsu, China) according to the manufacturer’s instructions. Equal amounts of brain tissue protein or CSF (20 μl) were separated by 10% sodium dodecyl sulfate-polyacrylamide gel electrophoresis and transferred to a polyvinylidene fluoride membrane (Millipore, Bedford, MA, United States). The membrane was then incubated with a primary antibody against Klotho (1:800; Transgene), retinoic acid-inducible gene I (RIG-I; 1:1000; Cell Signaling Technology, Beverly, MA, United States), β-actin (1:1000; Santa Cruz Biotechnology, Santa Cruz, CA, United States), NF-κB, or Lamin B (1:100; Boster) overnight at 4°C, followed by incubation with an appropriate horseradish peroxidase-conjugated secondary antibody (Zhongshan-Golden Bridge, Beijing, China) for 1 h at room temperature and an ECL chemiluminescence kit (Millipore, Billerica, MA, United States). The optical density (OD) of each band was determined using Gel Pro Analyzer 6.0 (Media Cybernetics, Bethesda, MD, United States). The immunoblot results were quantitatively analyzed densitometrically.

### Quantitative Real-time Polymerase Chain Reaction

To determine the mRNA levels of the target genes, total RNA was isolated from mouse brain tissues or the rat choroid plexus using Trizol reagent (Invitrogen Life Technologies, Carlsbad, CA, United States) and processed for cDNA, followed by real-time qPCR as described previously (Mao et al., 2017). The specific primer pairs (Beijing Genomics Institute, Beijing, China) are listed in **Table 1**. The mRNA levels of the target genes were normalized to GAPDH. The results are expressed as fold changes of the threshold cycle (*C*t) value relative to sham-operated controls using the 2^-ΔΔ^*^C^*_T_ method.

**Table 1:** The specific primer pairs used in polymerase chain reaction.

Gene	Forward	Reverse	Annealing temperature (°C)
Klotho (mice)	GGCTTTCCTCCTTTACCTGAAAA	CACATCCCACAGATAGACATTCG	54
TNF-α	CCCTCACACTCAGATCATCTTCT	GCTACGACGTGGGCTACAG	55
IL-6	CCAAGAGGTGAGTGCTTCCC	CTGTTGTTCAGACTCTCTCCCT	55
GAPDH	ACCACAGTCCATGCCATCAC	TCCACCACCCTGTTGCTGTA	54
Klotho (rat)	TAAGGTTCAAGTATGGAGAC	GGGCGTTCACACTTATTTAT	54
GAPDH	AGCGAGACCCCACTAACATC	GGTTCACACCCATCACAAAC	54

### Statistical Analysis

All of the data are expressed as mean values ± SEM. SPSS 19.0 software was used for the statistical analyses. Neurological scores and body weight data were analyzed by repeated-measures two-way ANOVA. *Post hoc* comparisons between groups were made using Dunnett’s T3 test (equal variances not assumed) or the Least Significant Difference test (equal variances assumed). The remaining data were analyzed by one-way ANOVA with Dunnett’s test. Values of *p* < 0.05 were considered statistically significant.

## Results

### Lentivirus System Upregulated Klotho Expression in 2VO Mice

The lentiviral vector system is a commonly used gene transfer approach. To explore the potential effects of Klotho on cerebral ischemic injury, our initial experiments first examined viral infectivity and Klotho overexpression that was induced by lentiviral particles under normal conditions. The lentiviral vectors had a C-terminal monomeric GFP tag, and viral infection was confirmed 72 h after transfection with lentiviral particles in HEK293 cells (**Supplementary Figure S1**). Two or 4 weeks after intracerebroventricular injection of the lentiviral suspension, LV-KL caused a nearly 100% increase in Klotho mRNA levels in the mouse choroid plexus compared with the LV-GFP and PBS groups (**Supplementary Figure S2**).

**FIGURE 1:**
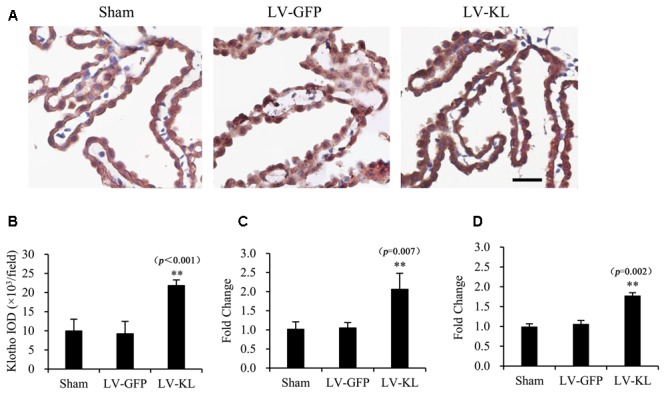
Overexpression of Klotho in the choroid plexus in 2VO mice induced by an intracerebroventricular injection of a lentivirus that encoded Klotho (LV-KL). Lentivirus that encoded green fluorescent protein (LV-GFP) was used as a vector control. Four weeks after intracerebroventricular injection of the lentiviral suspension, the mice were subjected to 20 min 2VO and 72 h reperfusion (LV-KL and LV-GFP groups) or LV-GFP-treated sham surgery (Sham group). Klotho expression in the choroid plexus was examined by immunohistochemistry and qPCR. **(A)** Representative immunohistochemical images showing that Klotho was successfully overexpressed using the lentivirus system in the choroid plexus. Scale bar = 50 μm. The cells that are representative of the overall staining intensity are magnified in the inset. **(B)** Quantitative image analysis of Klotho expression based on the integrated optical density (IOD) of positive immunostaining (brown) in the choroid plexus. **(C)** Quantitative analysis of Klotho mRNA levels by qPCR in the choroid plexus. **(D)** Quantitative analysis of Klotho mRNA levels by qPCR in the brain. The data are expressed as mean ± SEM. One-way ANOVA followed by Dunnett’s test. *n* = 4 or 5 per group. ^∗∗^*p* < 0.01, vs. LV-GFP-treated group.

Therefore, 4 weeks after intracerebroventricular transfection with the viral particles, the mice were subjected to 2VO, and immunohistochemistry and qPCR were performed to examine Klotho protein and mRNA levels. In the present study, no animals died from 20-min 2VO-induced forebrain ischemia and 72 h reperfusion. As shown in **Figure 1**, transient forebrain ischemia and reperfusion did not markedly affect Klotho protein or mRNA levels in the LV-GFP-treated 2VO group compared with sham-operated controls (*p* > 0.05), whereas LV-KL strongly upregulated Klotho expression in both the choroid plexus and ischemic brain (*p* < 0.01, vs. LV-GFP group).

### Klotho Overexpression Improved Neurological Outcomes in 2VO Mice

**FIGURE 2:**
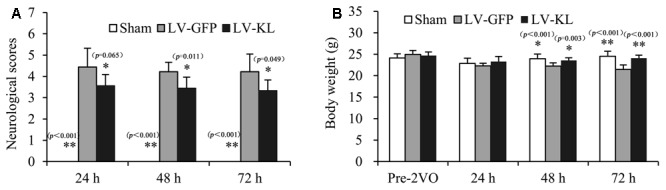
Effects of Klotho overexpression on neurological function in 2VO mice. Four weeks after the intracerebroventricular injection of a lentivirus that encoded Klotho (LV-KL) or GFP (LV-GFP), the mice were exposed to 20 min 2VO and 72 h reperfusion. **(A)** Neurobehavioral dysfunction (*F*_2,25_ = 34.120, *p* < 0.001) and **(B)** body weight data (*F*_1,24_ = 6.291, *p* < 0.01) were analyzed by repeated-measures two-way ANOVA followed by Dunnett’s T3 test or the Least Significant Difference test pre-2VO and 24, 48, and 72 h post-surgery. The data are expressed as mean ± SEM. *n* = 9 per group. ^∗^*p* < 0.05, ^∗∗^*p* < 0.01, vs. LV-GFP-treated 2VO group.

To explore the influence of Klotho on ischemic stress, we evaluated neurobehavioral function and body weight in lentivirus-treated 2VO mice every 24 h during a 72 h observation period after the onset of brain ischemia. As shown in **Figure 2A**, sham-operated mice exhibited no behavioral deficits and had a neurological deficit score of 0. Twenty minutes of forebrain ischemia resulted in neurobehavioral dysfunction, reflected by an increase in deficit scores in LV-GFP mice 24 h (4.44 ± 0.88), 48 h (4.22 ± 0.44), and 72 h (4.22 ± 0.83) after ischemia. LV-KL treatment effectively improved functional impairment at all of the tested times (**Figure 2A**). A significant difference was found in neurological scores among groups, and there is an overall difference between LV-GFP and LV-KL groups (*F*_2,25_ = 14.220, *p* < 0.001). The deficit scores were significantly lower in the LV-KL group 24 h (3.56 ± 0.53), 48 h (3.44 ± 0.53), and 72 h (3.33 ± 0.50) after 2VO compared with the LV-GFP group at the corresponding time points (all *p* < 0.05).

Additionally, in contrast to stable body weight in sham-operated mice during the experimental period, forebrain ischemia reduced body weight in LV-GFP-treated mice, and there is an overall difference between LV-GFP and LV-KL groups (*F*_2,25_ = 12.751, *p* < 0.001). At 48 and 72 h post-surgery, body weight in the LV-GFP group was significantly lower than in the sham group (*p* < 0.05 or *p* < 0.01, **Figure 2B**). The overexpression of Klotho significantly prevented the body weight reduction in the LV-KL group after 2VO (*p*.LV-GFP group; **Figure 2B**), which may be partially attributable to the improvement of neurobehavioral deficits.

### Klotho Overexpression Prevented Ischemic Brain Injury in 2VO Mice

**FIGURE 3:**
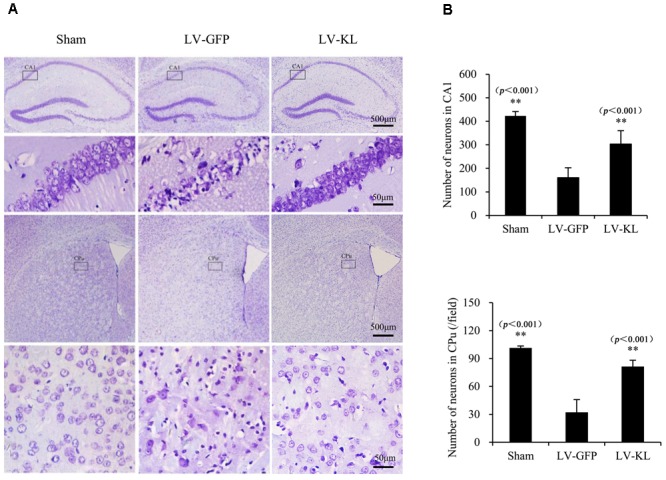
Effects of Klotho overexpression on ischemic brain injury in 2VO mice. Four weeks after the intracerebroventricular injection of a lentivirus that encoded Klotho (LV-KL) or GFP (LV-GFP), the mice were exposed to 20 min 2VO and 72 h reperfusion. Nissl staining was performed 72 h after surgery. **(A)** Representative images of Nissl staining showing ischemic lesions of neurons in the CA1 and caudate putamen (CPu) regions of the hippocampus. The cells that are representative of the 4× magnification field are magnified in the lower lane at 40× magnification. **(B)** Quantitative analysis of the number of normal neurons in the CA1 subregion and per field of the CPu (40×). *n* = 5 per group. The data are expressed as mean±SEM. One-way ANOVA followed by Dunnett’s test. ^∗∗^*p* < 0. 01, vs. LV-GFP- treated 2VO group.

To examine the effect of Klotho overexpression on cerebral ischemic injury, Nissl staining was used to detect surviving neurons in the same mice that were used for the immunohistochemistry analysis of Klotho immunoreactivity. As shown in **Figure 3**, forebrain ischemia significantly affected the number of hippocampal and striatal neurons in the LV-GFP group compared with sham controls. Seventy-two hours after 2VO, neuronal cell counts in both the hippocampal CA1 subfield and CPu per 40× field were significantly reduced in the LV-GFP group (160 ± 42 and 32 ± 14) compared with the sham group (423 ± 18 and 101 ± 2; *p* < 0.01, **Figure 3B**). The overexpression of Klotho effectively inhibited post-ischemia neuronal loss in the CA1 and CPu in the LV-KL group (302 ± 57 and 81 ± 7; *p* < 0.01, vs. LV-GFP group; **Figure 3B**).

### Klotho Overexpression Inhibited Neuropathological Changes in 2VO Mice

**FIGURE 4:**
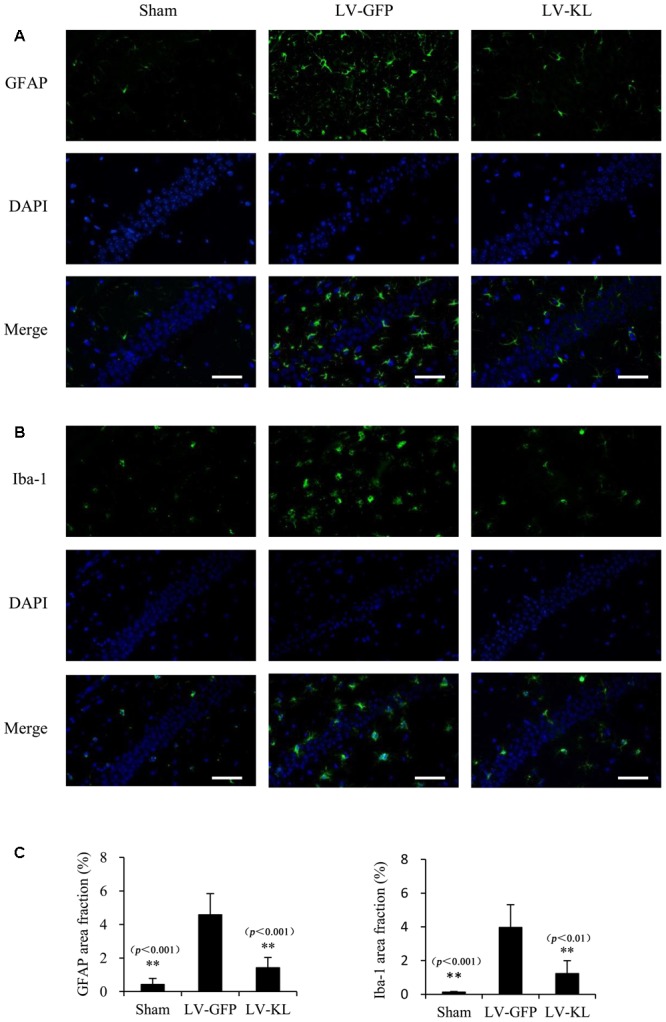
Effects of Klotho overexpression on neuroinflammatory responses in the ischemic brain induced by 2VO in mice. Four weeks after the intracerebroventricular injection of a lentivirus that encoded Klotho (LV-KL) or GFP (LV-GFP), the mice were exposed to 20 min 2VO and 72 h reperfusion. Immunofluorescent staining was performed 72 h after surgery. **(A)** Representative images of GFAP counterstained with nuclear DNA staining of 4, 6-diamidino- 2-phenylindole (DAPI) in the CA1 subregion. **(B)** Representative images of Iba-1 immunostaining in the CA1 subregion. **(C)** Quantitative image analysis of the immunoreactivity of GFAP and Iba-1based on the area fraction of GFAP or Iba-1-positive immunostaining in the CA1 area. The data are expressed as mean ± SEM. One-way ANOVA followed by Dunnett’s test. *n* = 5 per group. ^∗∗^*p* < 0.01, vs. LV-GFP-treated 2VO group. Scale bar = 50 μm.

Both activated residential glial cells and invading macrophages are implicated in the development of post-ischemic neuroinflammation and neuronal damage. GFAP and Iba-1 have been identified as specific markers of astrocyte and microglia/macrophage activation, respectively. In the present study, immunostaining for GFAP and Iba-1 was used to assess the effects of Klotho overexpression on the activation of these innate immune cells in the hippocampal CA1 subfield 72 h after cerebral hypoperfusion by 2VO. As shown in **Figure 4**, LV-GFP-treated 2VO mice exhibited significant increases in GFAP and Iba-1 immunoreactivity in the ischemic CA1 area compared with the sham-operated group (*p* < 0.01). Such immunoreactivity was markedly decreased by LV-KL treatment (*p* < 0.01). The present results suggest that Klotho overexpression in the brain significantly ameliorated neuroinflammatory neuropathological changes after cerebral ischemia.

### Klotho Overexpression Inhibited RIG-I/NF-κB Signaling in the Brain in 2VO Mice

**FIGURE 5:**
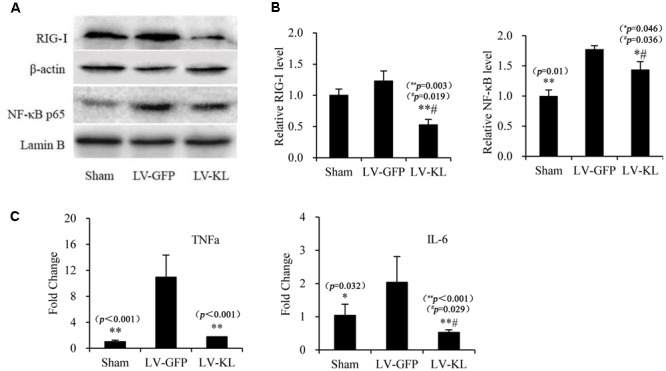
Effects of Klotho overexpression on neuroinflammatory responses in the ischemic brain induced by 2VO in mice. Four weeks after the intracerebroventricular injection of a lentivirus that encoded Klotho (LV-KL) or GFP (LV-GFP), the mice were exposed to 20 min 2VO and 72 h reperfusion. Western blot and qPCR were performed 72 h after surgery. **(A)** Representative Western blots and quantitative analysis of the expression of RIG-1 and the nuclear translocation of NF-κB. **(B)** The amount of RIG-I expression and NF-κB that translocated to the nucleus was normalized to β-actin or Lamin B, respectively. The results were expressed as each normalized value relative to LV-GFP-treated sham controls. **(C)** The mRNA levels of TNF-α and IL-6 in the brain. The results were normalized to the corresponding reporter gene GAPDH and are presented as a fold change relative to the sham-operated group. The data are expressed as mean ± SEM. One-way ANOVA followed by Dunnett’s test. *n* = 3–4 per group. ^∗^*p* < 0.05, ^∗∗^*p* < 0.01, vs. LV-GFP-treated 2VO group; ^#^*p* < 0.05, vs. LV-GFP-treated sham group.

To explore the mechanism that underlies Klotho overexpression-induced protection against cerebral hypoperfusion, we further evaluated the effects of LV-KL on the RIG-I/NF-κB pathway in the brain in 2VO mice. Western blot and qPCR were used to detect the activation RIG-I/NF-κB signaling and the production of downstream proinflammatory cytokines in the same mice that were used in the Klotho mRNA assay (**Figures 1C,D**, respectively). As shown in **Figure 5**, LV-KL significantly decreased RIG-I protein levels and tumor necrosis factor α (TNF-α) and interleukin 6 (IL-6) mRNA levels compared with the LV-GFP-treated 2VO group (*p* < 0.01) and significantly inhibited the nuclear translocation of NF-κB p65 compared with the LV-GFP-treated 2VO group (*p* < 0.05). Interestingly, LV-KL decreased RIG-I and IL-6 expression in ischemic brain tissues compared with the sham groups (*p* < 0.05). IL-6 has been reported to be one of the most important downstream proinflammatory cytokines that are regulated by RIG-I (Xia et al., 2016). The present results suggest that Klotho may effectively inhibit RIG-I/IL-6-induced inflammation, likely through both NF-κB-dependent and -independent mechanisms that are involved in brain ischemia.

### Klotho Knockdown Exacerbated Cerebral Ischemic Injury in MCAO Rats

**FIGURE 6:**
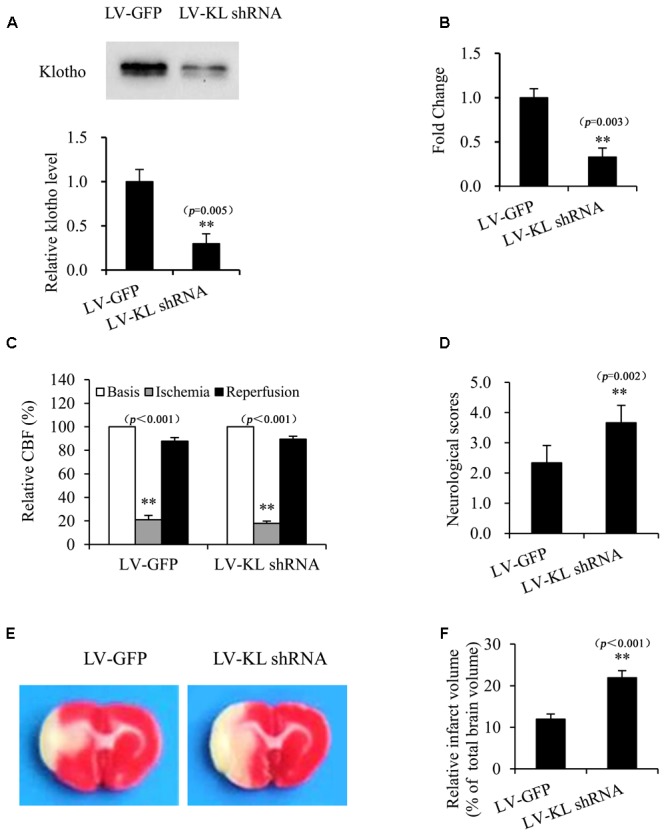
Klotho knockdown exacerbated cerebral ischemic injury in MCAO rats. One week after the intracerebroventricular injection of a lentivirus that harbored shRNA of Klotho (LV-KL shRNA) or control GFP (LV-GFP), cerebrospinal fluid **(A)** and the choroid plexus **(B)** were collected from normal rats for Western blot or qPCR, respectively. Additional rats were subjected to transient MCAO while monitoring cerebral blood flow (CBF) **(C)**. At 22 h of reperfusion after 2 h MCAO, neurobehavioral deficit scores were evaluated **(D)**, and the cerebral infarct volume was identified by TTC staining **(E)** and is expressed as a percentage of whole brain volume **(F)**. The data are expressed as mean ± SEM. One-way ANOVA followed by Dunnett’s test. *n* = 3 per group. ^∗∗^*p* < 0.01, vs. LV-GFP-treated group.

To further confirm the potential role of Klotho in resistance to cerebral ischemic, we investigated the effect of Klotho downregulation on focal cerebral ischemic injury that was induced by transient MCAO in rats. One week after the intracerebroventricular injection of the lentivirus that harbored shRNA of Klotho (LV-KL shRNA) or LV-GFP control, CSF and the choroid plexus were collected from normal rats to determine Klotho expression by Western blot and qPCR, respectively. The results indicated that Klotho content in CSF and Klotho mRNA levels in the choroid plexus decreased ∼70% in the LV-KL shRNA group compared with the LV-GFP group (*p* < 0.01; **Figures 6A,B**). Rats that were treated with LV-KL shRNA or LV-GFP were subjected to 2 h MCAO and 22 h reperfusion. LV-KL shRNA worsened neurobehavioral deficits and increased the infarct volume in ischemia/reperfusion rats compared with LV-GFP controls that were subjected to an equivalent ischemic challenge by monitoring CBF (*p* < 0.01; **Figures 6C–F**). These data show that Klotho knockdown exacerbated cerebral ischemic injury, suggesting that Klotho exerts an endogenous protective effect against brain ischemia.

## Discussion

Klotho is an antiaging gene, the expression of which declines with age, suggesting its potential role in the pathogenesis of aging-associated disorders. Our results showed that the lentivirus-mediated overexpression of Klotho significantly attenuated ischemic brain injury in mice, whereas Klotho knockdown that was induced by lentivirus-delivered shRNA exacerbated ischemic brain damage in rats. Moreover, Klotho overexpression significantly inhibited the post-ischemia inflammatory response in the brain, reflected by the inhibition of glia overactivation, inhibition of RIG-I/NF-κB signaling activation, and inhibition of proinflammatory cytokine production. These results provide direct evidence that Klotho may act as an endogenous neuroprotector against inflammatory damage that is caused by acute ischemic stroke.

Forebrain ischemia that is induced by transient 2VO is a useful mouse model of tolerance to brain ischemia (Bhuiyan et al., 2015), which closely recapitulates cerebral hypoperfusion that is caused by the progressive structural and functional changes in advanced arterial aging. In the present study, 4 weeks after lentivirus transfection with a bilateral intracerebroventricular injection of LV-KL (2.1 × 10^7^ TU/ml) in the lateral ventricles, the mice were subjected to brain ischemia that was induced by 20 min of 2VO, followed by collection of the choroid plexus to determine Klotho expression 72 h after ischemia. LV-KL successfully induced Klotho mRNA and protein overexpression in the choroid plexus and ischemic brain. The lentivirus-mediated overexpression of Klotho significantly improved neurological outcomes, improved neurobehavioral scores, recovered body weight, increased the number of surviving neurons, and attenuated activated microglia and reactive astrocytes in ischemic brain areas. Klotho overexpression significantly inhibited RIG-I expression, NF-κB activation, and proinflammatory cytokine (TNF-α and IL-6) production in ischemic brain tissues compared with LV-GFP controls. These findings suggest that Klotho overexpression may protect against ischemic brain damage at least partially through the inhibition of RIG-I/NF-κB inflammatory signaling.

Interestingly, our results showed that Klotho overexpression exerted different inhibitory effects on diverse inflammatory markers that are involved in the RIG-I/NF-κB signaling pathway. Notably, LV-KL significantly reduced RIG-I and IL-6 levels in the ischemic brain in 2VO mice compared with sham-operated controls (*p* < 0.05). RIG-I is a caspase recruitment domain (CARD)-containing protein that contains two N-terminal CARD-like domains and a C-terminal RNA helicase domain (Yoneyama et al., 2004). RIG-I is generally regarded as an important innate immune sensor that recognizes viral RNA in the cytoplasm and enhances antiviral host defense (Seth et al., 2005; Poeck et al., 2010). RIG-I may also play an important role in both acute inflammation in lipopolysaccharide-stimulated macrophages and chronic inflammation in replicative senescent human umbilical cord vein endothelial cells (HUVECs) through the production of various potent proinflammatory cytokines, such as IL-6 (Wang et al., 2008; Liu et al., 2011). Senescence can induce RIG-I inflammation by inducing its expression and multimerization, and Klotho may block RIG-I multimerization and subsequent activation through a direct interaction with the CARD domain of RIG-I (Liu et al., 2011). Our previous study found that both aging-induced RIG-I expression and Klotho decline are associated with chronic inflammation in the kidney in aged senescence-accelerated mice (Zeng et al., 2016). The present study found that Klotho overexpression induced by LV-KL directly inhibited RIG-I expression in the ischemic brain. Additionally, a recent study showed that Klotho gene overexpression significantly decreased IL-6 production in HUVECs with or without inflammatory challenge (Xia et al., 2016), and IL-6 receptor polymorphisms contributed to neurological status in ischemic stroke patients (Kim et al., 2016). The binding of IL-6 to the IL-6 receptor activates intracellular signaling cascades and the production of C-reactive protein (CRP) and other cytokines (Wang et al., 2010). Notably, IL-6 has been reported to function as a main stimulator of CRP that predicts clinical stroke risk (Ridker et al., 2002). Therefore, our results provide further direct evidence of the therapeutic potential of Klotho in conferring resistance to RIG-I/IL-6 signaling-associated disorders, such as cerebral ischemic insult.

In the present study, we also used a rat model of transient focal cerebral ischemia to explore the potential role of Klotho in the resistance to cerebral ischemia. Rats were used because of the ease with which both the cerebral choroid plexus and CSF can be collected. Furthermore, the experimental rat model more closely resembles clinical acute ischemic stroke. We found that LV-KL-shRNA effectively downregulated the mRNA levels of transmembrane Klotho in the choroid plexus and decreased the release of ectodomain protein levels of Klotho in CSF 1 week after intracerebroventricular administration of lentiviral particles compared with control LV-GFP, indicating a significant reduction of the anti-inflammatory intracellular form of Klotho. We then investigated differences between rats with Klotho knockdown by LV-KL-shRNA and LV-GFP-treated control rats in their response to an equivalent ischemic insult by monitoring CBF. One week after administration, LV-KL-shRNA exacerbated cerebral ischemic injury, reflected by increases in both neurological deficit scores and infarct volume, revealed by TTC staining. Our results provide direct evidence that the age-related decline of Klotho may contribute to acute ischemic stroke and poor outcomes in the elderly.

The pleiotropic effects of Klotho during aging have been gradually revealed. Klotho has become a promising therapeutic target for aging-related disorders, including neurodegeneration and stroke. Klotho itself and small-molecule Klotho enhancers have been tested in high-throughput screening, cultured cells, and animal models (Abraham et al., 2012; King et al., 2012a; Kuang et al., 2014a; Zeldich et al., 2014). Our previous study found that ligustilide is as a natural phthalide enhancer of Klotho in both cultured cells and mice (Kuang et al., 2014a). Ligustilide also inhibited neuroinflammatory responses in various animal models of brain ischemia (Kuang et al., 2006, 2008, 2014b). These data provide evidence of the therapeutic potential of small-molecular enhancers of Klotho in ischemic stroke.

## Conclusion

The present findings indicate that the antiaging Klotho may act as an endogenous neuroprotective factor against cerebral ischemic injury, at least partially by inhibiting RIG-I/NF-κB inflammatory signaling. Klotho itself or enhancers of Klotho may compensate for aging-related decline and may be a promising approach for preventing and treating acute ischemic stroke during advanced age. Further studies will be conducted to examine whether Klotho hyperexpression has effects on glia and cytokines in normal brain.

## Author Contributions

Study design: J-RD. Experiments and data analysis: H-JZ, HL, M-QS, X-NM, J-RD, D-LL, Y-RC, Y-MG, and XK. Study supervision and paper writing: J-RD. Final approval of the version to be published: All authors.

## Conflict of Interest Statement

The authors declare that the research was conducted in the absence of any commercial or financial relationships that could be construed as a potential conflict of interest.

## Supplementary Material

FIGURE S1 Viral infectivity induced by a lentivirus that encoded *GFP* or Klotho (LV-GFP or LV-KL) in HEK293 cells. Seventy-two hours after transfection with vehicle or lentiviral particles, fluorescent microscopy and light microscopy results showed that the transfection efficiency of both LV-GFP and LV-KL was more than 70% *in vitro*. Scale bar = 50 μm.

FIGURE S2 Lentivirus that encoded Klotho (LV-KL) induced Klotho overexpression in the choroid plexus in normal mice. Two or 4 weeks after the intracerebroventricular injection of PBS or the lentiviral suspension (LV-KL or LV-GFP), the choroid plexus was collected from mice for the quantitative analysis of Klotho mRNA levels by qPCR. The results are expressed as mean ± SEM. One-way ANOVA followed by Dunnett’s test. *n* = 3/group. ^∗∗^*p* < 0.01, vs. LV-GFP group.

## References

[B1] Abraham C. R. Chen C. D. Cuny G. D. Glicksman M. A. Zeldich E. (2012). Small-molecule Klotho enhancers as novel treatment of neurodegeneration. *Future Med. Chem.* 4 1671–1679. 10.4155/fmc.12.134 22924505 PMC3564652

[B2] Aurel P. W. Stanley-Thomas C. Zaal K. Christof K. Lary C. W. (2007). The response of the aged brain to stroke: too much, too soon? *Curr. Neurovasc. Res.* 4 216–227. 10.2174/156720207781387213 17691975

[B3] Bhuiyan M. I. H. Kim J. C. Hwang S. N. Lee M. Y. Kim S. Y. (2015). Ischemic tolerance is associated with VEGF-C and VEGFR-3 signaling in the mouse hippocampus. *Neuroscience* 290 90–102. 10.1016/j.neuroscience.2015.01.025 25637798

[B4] Bolch L. Sineshchekowa O. Reichenbach D. Reiss K. Saffig P. Kuro-o M. (2009). Klotho is a substrate for alpha-, beta and gamma-secretase. *FEBS Lett.* 583 3221–3224. 10.1016/j.febslet.2009.09.009 19737556 PMC2757472

[B5] Calabrese V. Guagliano E. Sapienza M. Panebianco M. Calafato S. Puleo E. (2007). Redox regulation of cellular stress response in aging and neurodegenerative disorders: role of vitagenes. *Neurochem. Res.* 32 757–773. 10.1007/s11064-006-9203-y 17191135

[B6] Chen C. D. Podvin S. Gillespie E. Leeman S. E. Abraham C. R. (2007). Insulin stimulates the cleavage and release of the extracellular domain of Klotho by ADAM10 and ADAM17. *Proc. Natl. Acad. Sci. U.S.A.* 104 19796–19801. 10.1073/pnas.0709805104 18056631 PMC2148378

[B7] DiNapoli V. A. Huber J. D. Houser K. Li X. Rosen C. L. (2008). Early disruptions of the blood-brain barrier may contribute to exacerbated neuronal damage and prolonged functional recovery following stroke in aged rats. *Neurobiol. Aging* 29 753–764. 10.1016/j.neurobiolaging.2006.12.007 17241702 PMC2683361

[B8] Duce J. A. Podvin S. Hollander W. Kipling D. Rosene D. L. Abraham C. R. (2008). Gene profile analysis implicates Klotho as an important contributor to aging changes in brain white matter of the rhesus monkey. *Glia* 56 106–117. 10.1002/glia.20593 17963266

[B9] Imura A. Iwano A. Tohyama O. Tsuji Y. Nozaki K. Hashimoto N. (2004). Secreted Klotho protein in sera and CSF: implication for post-translational cleavage in release of Klotho protein from cell membrane. *FEBS Lett.* 565 143–147. 10.1016/j.febslet.2004.03.090 15135068

[B10] Kim D. H. Yoo S. D. Chon J. Yun D. H. Kim H. S. Park H. J. (2016). Interleukin-6 receptor polymorphisms contribute to the neurological status of Korean patients with ischemic stroke. *J. Korean Med. Sci.* 31 430–434. 10.3346/jkms.2016.31.3.430 26955245 PMC4779869

[B11] Kim Y. Kim J. H. Nam Y. J. Kong M. Kim Y. J. Yu K. H. (2006). Klotho is a genetic risk factor for ischemic stroke caused by cardioembolism in Korean females. *Neurosci. Lett.* 407 189–194. 10.1016/j.neulet.2006.08.039 16973281

[B12] King G. D. Chen C. D. Huang M. M. Zeldich E. Brazee P. L. Schuman E. R. (2012a). Identification of novel small molecules that elevate Klotho expression. *Biochem. J.* 441 453–461. 10.1042/BJ20101909 21939436 PMC3677209

[B13] King G. D. Rosene D. L. Abraham C. R. (2012b). Promoter methylation and age-related downregulation of Klotho in rhesus monkey. *Age* 34 1405–1419. 10.1007/s11357-011-9315-4 21922250 PMC3528360

[B14] Kuang X. Chen Y. S. Wang L. F. Li Y. J. Liu K. Zhang M. X. (2014a). Klotho upregulation contributes to the neuroprotection of ligustilide in an Alzheimer’s disease mouse model. *Neurobiol. Aging* 35 169–178. 10.1016/j.neurobiolaging.2013.07.019 23973442

[B15] Kuang X. Du J. R. Chen Y. S. Wang J. Wang Y. N. (2009). Protective effect of Z-ligustilide against amyloid beta-induced neurotoxicity is associated with decreased pro-inflammatory markers in rat brains. *Pharmacol. Biochem. Behav.* 92 635–641. 10.1016/j.pbb.2009.03.007 19324070

[B16] Kuang X. Du J. R. Liu Y. X. Zhang G. Y. Peng H. Y. (2008). Postischemic administration of Z-Ligustilide ameliorates cognitive dysfunction and brain damage induced by permanent forebrain ischemia in rats. *Pharmacol. Biochem. Behav.* 88 213–221. 10.1016/j.pbb.2007.08.006 17889286

[B17] Kuang X. Wang L. F. Yu L. Li Y. J. Wang Y. N. He Q. (2014b) Ligustilide ameliorates neuroinflammation and brain injury in focal cerebral ischemia/reperfusion rats: involvement of inhibition of TLR4/peroxiredoxin 6 signaling. *Free Radic. Biol. Med.* 71 165–175. 10.1016/j.freeradbiomed.2014.03.028 24681253

[B18] Kuang X. Yao Y. Du J. R. Liu Y. X. Wang C. Y. Qian Z. M. (2006). Neuroprotective role of Z-ligustilide against forebrain ischemic injury in ICR mice. *Brain Res.* 1102 145–153. 10.1016/j.brainres.2006.04.110 16806112

[B19] Kuro-o M. Matsumura Y. Aizawa H. Kawaguchi H. (1997). Mutation of the mouse Klotho gene leads to a syndrome resembling ageing. *Nature* 390 45–51. 10.1038/36285 9363890

[B20] Kurosu H. Yamamoto M. Clark J. D. Pastor J. V. Nandi A. Gurnani P. (2005). Suppression of aging in mice by the hormone Klotho. *Science* 309 1829–1833. 10.1126/science.1112766 16123266 PMC2536606

[B21] Li L. Wang Y. Gao W. Yuan C. Zhang S. Zhou H. (2015). Klotho reduction in alveolar macrophages contributes to CSE-induced inflammation in chronic obstructive pulmonary disease. *J. Biol. Chem.* 290 27890–27900. 10.1074/jbc.M115.655431 26385922 PMC4646031

[B22] Liu F. Wu S. Ren H. Gu J. (2011). Klotho suppresses RIG-I-mediated senescence- associated inflammation. *Nat. Cell Biol.* 13 254–262. 10.1038/ncb2167 21336305

[B23] Maekawa Y. Ishikawa K. Yasuda O. Oguro R. Hanasaki H. Kida I. (2009). Klotho suppresses TNF-alpha-induced expression of adhesion molecules in the endothelium and attenuates NF-kappaB activation. *Endocrine* 35 341–346. 10.1007/s12020-009-9181-3 19367378

[B24] Mao X. N. Zhou H. J. Yang X. J. Zhao L. X. Kuang X. Chen C. (2017). Neuroprotective effect of a novel gastrodin derivative against ischemic brain injury: involvement of peroxiredoxin and TLR4 signaling inhibition. *Oncotarget* 8 90979–9099. 10.18632/oncotarget.18773 29207618 PMC5710899

[B25] Marutani E. Kosugi S. Tokuda K. Khatri A. Nguyen R. Atochin D. N. (2012). A novel hydrogen sulfide-releasing *N*-methyl-D-aspartate receptor antagonist prevents ischemic neuronal death. *J. Biol. Chem.* 287 32124–32135. 10.1074/jbc.M112.374124 22815476 PMC3442543

[B26] Mozaffarian D. Benjamin E. J. Go A. S. Arnett D. K. Blaha M. J. Cushman M. (2015). Heart disease and stroke statistics-2015 update: a report from the American Heart Association. *Circulation* 131 e29–e322. 10.1161/CIR.0000000000000157 25520374

[B27] Pedersen L. Pedersen S. M. Brasen C. L. Rasmussen L. M. (2013). Soluble serum Klotho levels in healthy subjects comparison of two different immunoassays. *Clin. Biochem.* 46 1079–1083. 10.1016/j.clinbiochem.2013.05.046 23707222

[B28] Poeck H. Bscheider M. Gross O. Finger K. Roth S. Rebsamen M. (2010). Recognition of RNA virus by RIG-I results in activation of CARD9 and inflammasome signaling for interleukin 1 beta production. *Nat. Immunol.* 11 63–69. 10.1038/ni.1824 19915568

[B29] Ridker P. M. Rifai N. Rose L. Buring J. E. Cook N. R. (2002). Comparison of C-reactive protein and low-density lipoprotein cholesterol levels in the prediction of first cardiovascular events. *N. Engl. J. Med.* 347 1557–1565. 10.1056/NEJMoa021993 12432042

[B30] Rosen C. L. Dinapoli V. A. Nagamine T. Crocco T. (2005). Influence of age on stroke outcome following transient focal ischemia. *J. Neurosurg.* 103 687–694. 10.3171/jns.2005.103.4.0687 16266051

[B31] Seth R. B. Sun L. Ea C. K. Chen Z. J. (2005). Identification and characterization of MAVS, a mitochondrial antiviral signaling protein that activates NF-κB and IRF 3. *Cell* 122 669–682. 10.1016/j.cell.2005.08.012 16125763

[B32] Shen J. Bai X. Y. Qin Y. Jin W. W. Zhou J. Y. Zhou J. P. (2011). Interrupted reperfusion reduces the activation of NADPH oxidase after cerebral I/R injury. *Free Radic. Biol. Med.* 50 1780–1786. 10.1016/j.freeradbiomed.2011.03.028 21458562

[B33] Troen B. R. (2003). The biology of aging. *Mt. Sinai J. Med.* 70 3–22. 12516005

[B34] Wang J. Wu S. Jin X. Li M. Chen S. Teeling J. L. (2008). Retinoic acid-inducible gene-I mediates late phase induction of TNF-àby lipopolysaccharide. *J. Immunol.* 180 8011–8019. 10.4049/jimmunol.180.12.8011 18523264

[B35] Wang M. Song H. Jia J. (2010). Interleukin-6 receptor gene polymorphisms were associated with sporadic Alzheimer’s disease in Chinese Han. *Brain Res.* 1327 1–5. 10.1016/j.brainres.2010.02.067 20197062

[B36] Xia W. Zhang A. Jia Z. Gu J. Chen H. (2016). Klotho contributes to pravastatin effect on suppressing il-6 production in endothelial cells. *Mediators Inflamm.* 2016:2193210. 10.1155/2016/2193210 27034587 PMC4789490

[B37] Yamazaki Y. Imura A. Urakawa I. Shimada T. Murakami J. Aano Y. (2010). Establishment of sandwich ELISA for soluble alpha-Klotho measurement: age-dependent change of soluble alpha-Klotho levels in healthy subjects. *Biochem. Biophys. Res. Commun.* 398 513–518. 10.1016/j.bbrc.2010.06.110 20599764 PMC4130489

[B38] Yoneyama M. Kikuchi M. Natsukawa T. Shinobu N. Imaizumi T. Miyagishi (2004). The RNA helicase RIG-I has an essential function in double-stranded RNA induced innate antiviral responses. *Nat. Immunol.* 5 730–737. 10.1038/ni1087 15208624

[B39] Zeldich E. Chen C. D. Colvin T. A. Bove-Fenderson E. A. Liang J. Zhou T. B. T. (2014). The neuroprotective effect of Klotho is mediated via regulation of members of the redox system. *J. Biol. Chem.* 289 24700–24715. 10.1074/jbc.M114.567321 25037225 PMC4148892

[B40] Zeng Y. Wang P. H. Zhang M. Du J. R. (2016). Aging-related renal injury and inflammation are associated with downregulation of Klotho and induction of RIG-I/NF-κB signaling pathway in senescence-accelerated mice. *Aging Clin. Exp. Res.* 28 69–76. 10.1007/s40520-015-0371-y 25986237

