# Supplementary figures and images for: Protective Effect of Klotho against Ischemic Brain Injury Is Associated with Inhibition of RIG-I/NF-κB Signaling
Source: Front Pharmacol. 2018 Jan 18;8:950. doi: 10.3389/fphar.2017.00950 (PMC5778393; doi:10.3389/fphar.2017.00950)

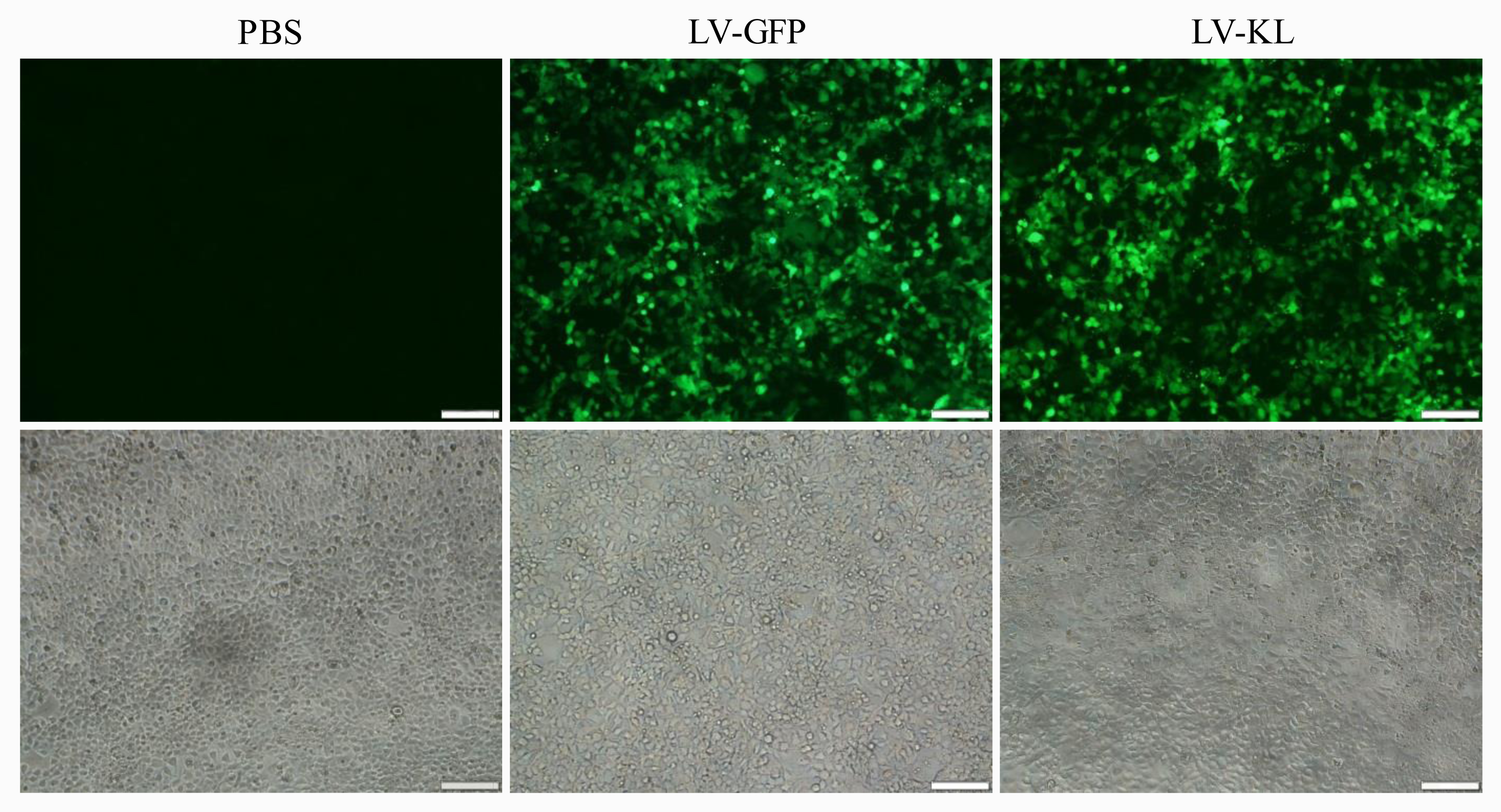

Supplement: FIGURE S1 — Viral infectivity induced by a lentivirus that encoded GFP or Klotho (LV-GFP or LV-KL) in HEK293 cells. Seventy-two hours after transfection with vehicle or lentiviral particles, fluorescent microscopy and light microscopy results showed that the transfection efficiency of both LV-GFP and LV-KL was more than 70% in vitro. Scale bar = 50 μm. [file Image_1.TIF]

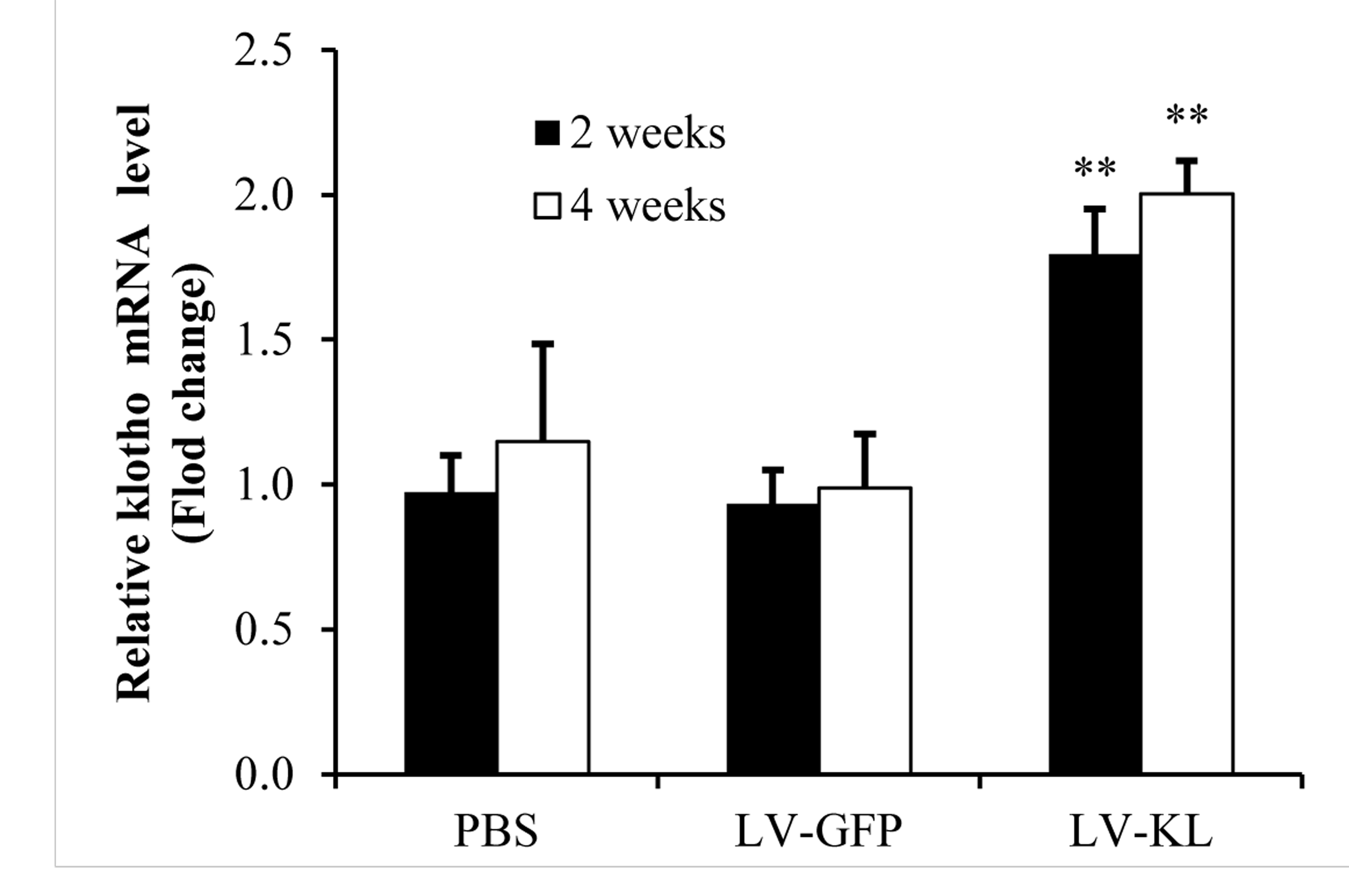

Supplement: FIGURE S2 — Lentivirus that encoded Klotho (LV-KL) induced Klotho overexpression in the choroid plexus in normal mice. Two or 4 weeks after the intracerebroventricular injection of PBS or the lentiviral suspension (LV-KL or LV-GFP), the choroid plexus was collected from mice for the quantitative analysis of Klotho mRNA levels by qPCR. The results are expressed as mean ± SEM. One-way ANOVA followed by Dunnett’s test. n = 3/group. ∗∗p < 0.01, vs. LV-GFP group. [file Image_2.TIF]
